# *Streptococcus gordonii*: A Rare Cause of Infective Endocarditis

**DOI:** 10.1155/2019/7127848

**Published:** 2019-06-12

**Authors:** Natalia Mosailova, Justina Truong, Tyson Dietrich, John Ashurst

**Affiliations:** ^1^Pacific Northwest University of Health Sciences, 111 University Parkway, Suite 202, Yakima, WA 98901, USA; ^2^Kingman Regional Medical Center, Department of Emergency Medicine, 3269 Stockton Hill Road, Kingman, AZ 86409, USA; ^3^Kingman Regional Medical Center, Department of Pharmacy, 3269 Stockton Hill Road, Kingman, AZ 86409, USA

## Abstract

Infective endocarditis is a rare but life-threatening disease seen across the globe. Organisms from the oral cavity still represent a large proportion of pathogens seen in endocarditis and can be from either daily dental routines or invasive procedures. With the recent changes to antibiotic prophylaxis for infective endocarditis prior to dental procedures, the physician must have a heightened degree of suspicion when presented with a patient with undifferenced sepsis following dental procedures. The authors present a case of infective endocarditis caused by *Streptococcus gordonii* after the drainage of a dental abscess.

## 1. Introduction

Infective endocarditis (IE) is a rare but devastating illness faced by both low- and high-income nations alike. A link between IE and dental procedures has been identified and occurs via the breakdown of the mucocutaneous barriers. The viridans group of streptococci (VGS) are bacteria that have a low virulence and are usually present in the oral cavity, upper airways, gastrointestinal tract, and female genitalia [1]. The group is further classified into six major subgroups: *S. mutans* group, *S. mitis* group, *S. anginosus* group, *S. salivarius* group, *S. bovis* group, and *S. sanguinis* group [1, 2]. The *S. sanguinis* group includes the bacteria *S*. *sanguinis*, *S*. *parasanguinis*, and *S. gordonii* and rarely causes invasive infections including IE [1, 2]. The authors present a case of infective endocarditis caused by *S. gordonii* in an immunocompetent patient following a dental procedure.

## 2. Case

A 31-year-old male presented to the emergency department secondary to bilateral lower extremity edema for the last two days. He denied shortness of breath, chest pain, fever, and chills. The patient's past medical history was unremarkable except for a recent dental procedure to drain abscess. His social history was significant for recent alcohol abuse and denied intravenous drug use.

Physical exam showed a temperature of 102.8 F, heart rate of 154 beats per minute, respiratory rate of 20 breaths per minute, and an oxygen saturation of 94% on room air. The cardiovascular exam revealed a normal rhythm with tachycardia but no murmurs, rubs, or gallops. He also had rales present to the mid lungs bilaterally without wheezes or rhonchi. Extremity examination revealed +1 pitting edema of the bilateral lower extremities and clubbing of the fingernails bilaterally without lesions or rashes on the palms or soles of the feet.

While in the emergency department, labs revealed a white blood cell count of 10.7 K/uL (4.8–10.8 K/uL), hemoglobin of 7.6 g/dL (13.1–17.1 g/dL), hematocrit of 23.6% (42.0–52.0%), platelet of 315 K/uL (150–450 K/uL), lactate of 1.5 mmol/L (0.7–2.0 mmol/L), procalcitonin of 3.11 ng/mL (0.05–1.90 ng/mL), pro-BNP of 1720 pg/mL (0–125 pg/mL), and a troponin of 0.131 ng/mL (0.000–0.034 ng/mL). The chest radiograph showed no signs of infection or pulmonary edema. Computed tomography of the chest, abdomen, and pelvis with intravenous contrast showed an atypical inflammatory pattern in the lungs with mild cardiomegaly, hepatomegaly, and splenomegaly with a hypodensity suggestive of an infarct.

The patient was started on empiric antibiotics of piperacillin/tazobactam and vancomycin, normal saline bolus of 30 cc/kg, and two units of packed red blood cells and admitted to the transitional care unit for further management of sepsis due to an unclear etiology. While on the floor, the patient became hypoxic and required noninvasive ventilation and was transitioned to intensive care unit. The following morning, a transthoracic echocardiogram revealed a medium-sized mobile vegetation on the body of the anterior leaflet of the mitral valve and severe mitral valve regurgitation. Both blood cultures were positive for *S. gordonii*, and antibiotics were deescalated to ceftriaxone and vancomycin based upon sensitivity analysis. The patient was transferred to a tertiary care center where he underwent a mechanical mitral valve replacement and was placed on ceftriaxone for six weeks.

## 3. Discussion

IE is defined as an “infection of a native or prosthetic heart valve, the endocardial surface, or an indwelling cardiac device” [3]. The disease is relatively rare worldwide with an annual incidence of 3–10 cases per 100,000 people but carries a short-term mortality of 10 to 30% [3, 4]. Recently, a shift has been seen in the epidemiology of those inflicted with the disease [3]. In the early microbial era, the epidemiology in low- and high-income countries were mirrored by the risk factor of rheumatic heart disease causing IE to be prevalent in young adults [3]. As the incidence of rheumatic heart disease decreased in high-income countries, an epidemiological shift occurred in which the elderly (age over 65) are now more likely to contract the disease [3].

*Staphylococcus*, *Streptococcus*, and *Enterococcus* species account for between 80 and 90% of all cases of IE worldwide [3]. The VGS is the most common cause of IE in low-income countries and is one of the most prevalent bacteria in the oral cavity [3]. However, *S. gordonii* is a rare cause of IE that has been sparsely reported in the literature. *S. gordonii* are Gram-positive,alpha-hemolytic chains of cocci that play an important role in the alkalization of the oral cavity and protective biofilm production [2]. Once in the blood stream, *S. gordonii* appear to have the virulence factors that are pathogenic in the development of IE. The cell wall of *S*. *gordonii* contains a serine-rich glycoprotein, GspB, which mediates binding to human platelets [5]. After adherence to the platelet, the combination has the potential to attach to the fibronectin-rich extracellular matrix of the cardiac valves and subsequently form valvular vegetations [5].

For almost half a century, oral antibiotic prophylaxis was given to those deemed at risk of developing IE prior to dental procedures [6]. In 1997, the American Heart Association (AHA) noted that most cases of IE were not related to invasive dental procedures but instead due to daily activities such as tooth brushing and chewing [6]. In 2007, the AHA changed their recommendations to ongoing prophylaxis only for those patients at highest risk of developing IE (those with history of infective endocarditis, prosthetic valves, and cardiac transplantation who develop cardiac valvulopathy and congenital heart disease) [7]. Then, in 2008, the United Kingdom (UK) National Institute for Health and Clinical Excellence advised for the cessation of antibiotic prophylaxis for endocarditis prior to dental procedures citing the lack of strong clinical evidence, the overall low risk of endocarditis arising from dental procedures, and the indiscriminate use of antibiotics [8]. Since the cessation of antibiotic prophylaxis for IE in the United Kingdom, a small (0.11 cases per 10 million people per month) but statistically significant increase in cases of IE has been noted [9]. Based upon these results, further studies need to be undertaken which involve a placebo-controlled, double blind study to determine the efficacy of IE prophylaxis prior to dental procedures.

## 4. Conclusion

Although a rare entity, IE should be included in the physician's differential in those patients with undifferentiated sepsis following dental procedures. A high degree of suspicion is needed to prevent both the relatively high rates of morbidity and mortality associated with IE.
